# Supplementary figures and images for: A dynamic population of prophase CENP-C is required for meiotic chromosome segregation
Source: PLoS Genet. 2023 Nov 29;19(11):e1011066. doi: 10.1371/journal.pgen.1011066 (PMC10721191; doi:10.1371/journal.pgen.1011066)

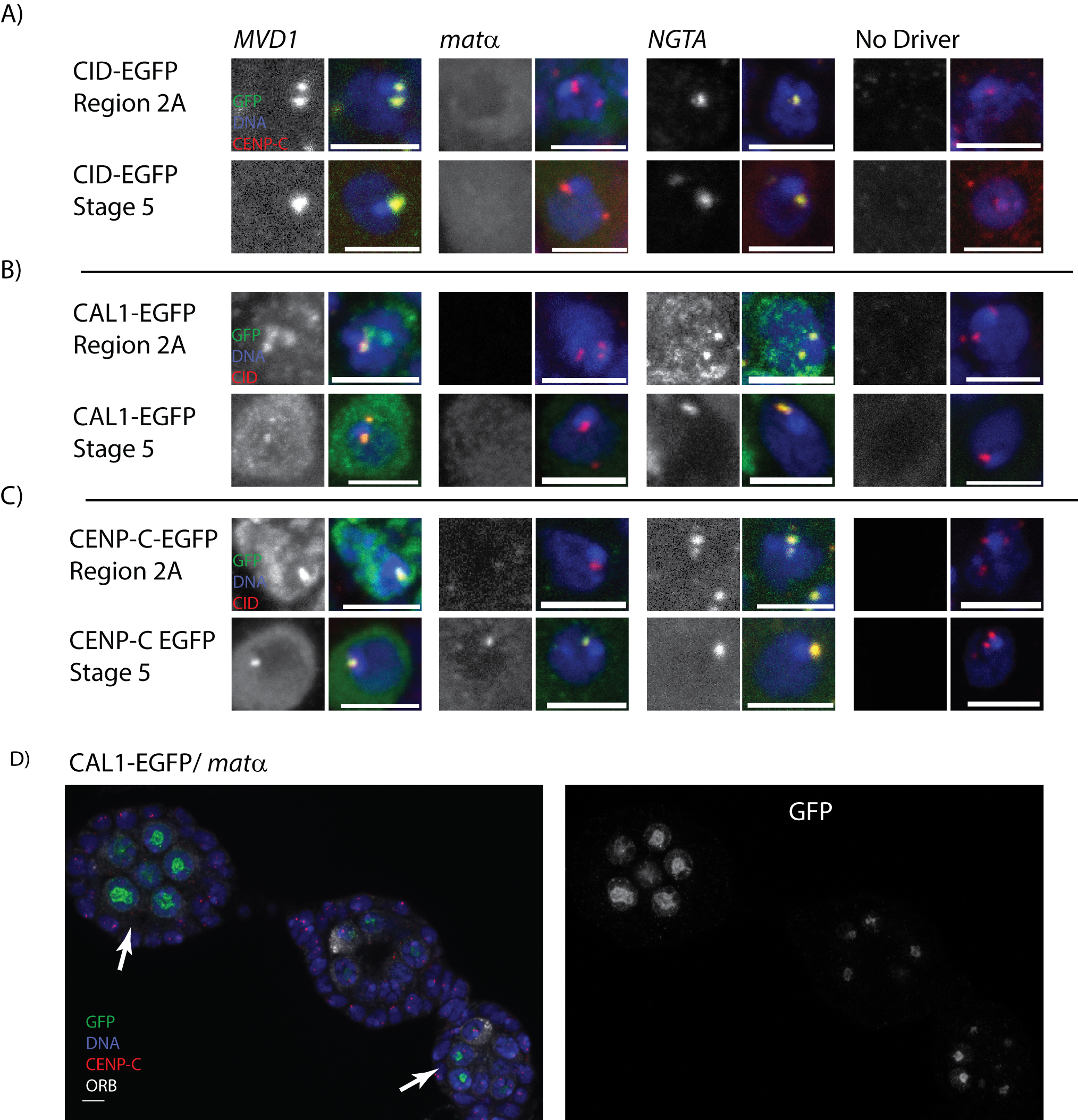

Supplement: S1 Fig — A) Localization of GFP-CID (green) and centromeres detected using a CENP-C antibody (red). B) Localization of GFP-CAL1 (green) with the centromeres detected using a CID antibody (red). C) Localization of GFP-CENP-C, with the centromeres detected using a CID antibody (red). In all images, the DNA is blue and the scale bars are 5 μm. D) Localization of GFP-CAL1 (green) using matα in early stages of the vitellarium (stages 1–5). GFP-CAL1 protein can been seen accumulating in nurse cell nuclei (arrows), but not at the centromeres in the oocyte. The centromeres were detected using a CENP-C antibody (red) and the cytoplasmic ORB protein (white) is enriched in the oocyte. The scale bar is 10 μm. (TIF) [file pgen.1011066.s001.tif]

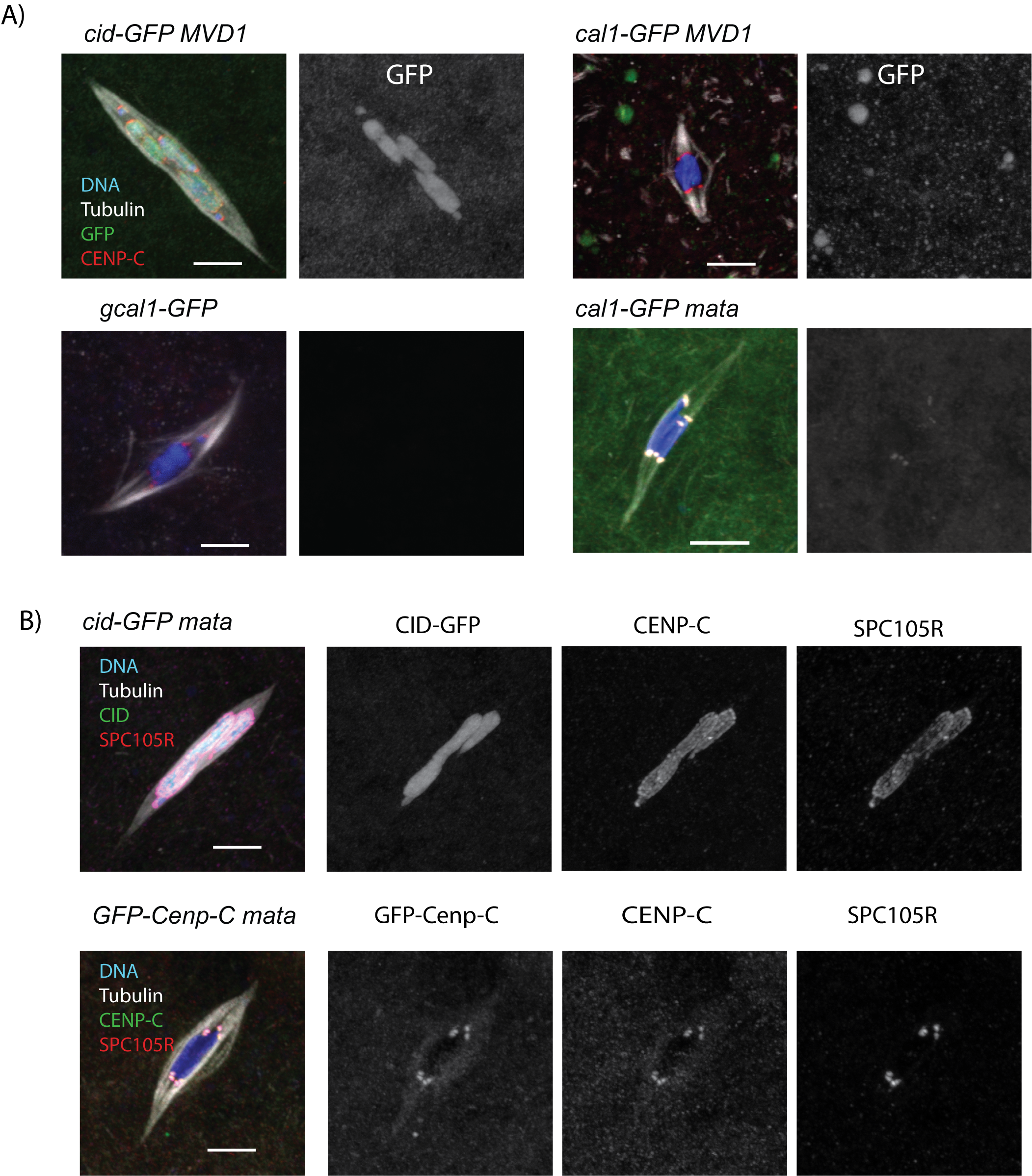

Supplement: S2 Fig — The DNA is blue, microtubules in white, and the scale bars represent 5 μm. A) Localization of CID-GFP or CAL1-GFP (green) using the MVD1 or matα. In the one image (gcal1-GFP), CAL1-GFP is regulated by the endogenous cal1 promoter. The centromeres were detected using CENP-C (red). B) Localization of CID-GFP or GFP-CENP-C (green) using matα. The kinetochores were detected using an antibody against Spc105R (red). (TIF) [file pgen.1011066.s002.tif]

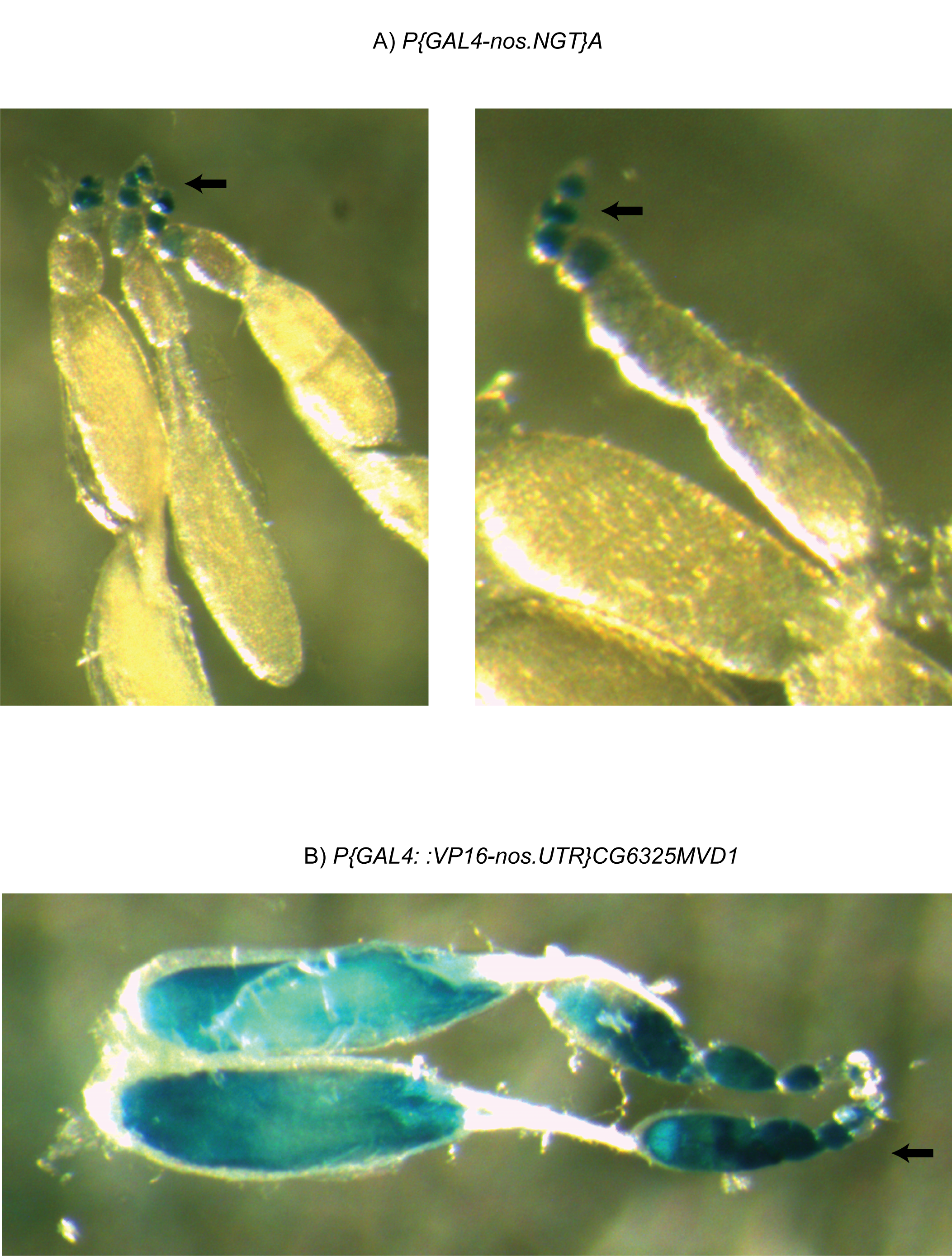

Supplement: S3 Fig — The expression pattern of (A) P{GAL4-nos.NGT} and (B) P{GAL4::VP16-nos.UTR}CG6325MVD1 using UASP-β-galactosidase as a reporter. Arrowheads indicate anterior tip of the ovariole, where the germarium is located, and the blue stain indicates where each GAL4 promotes expression. (TIF) [file pgen.1011066.s003.tif]

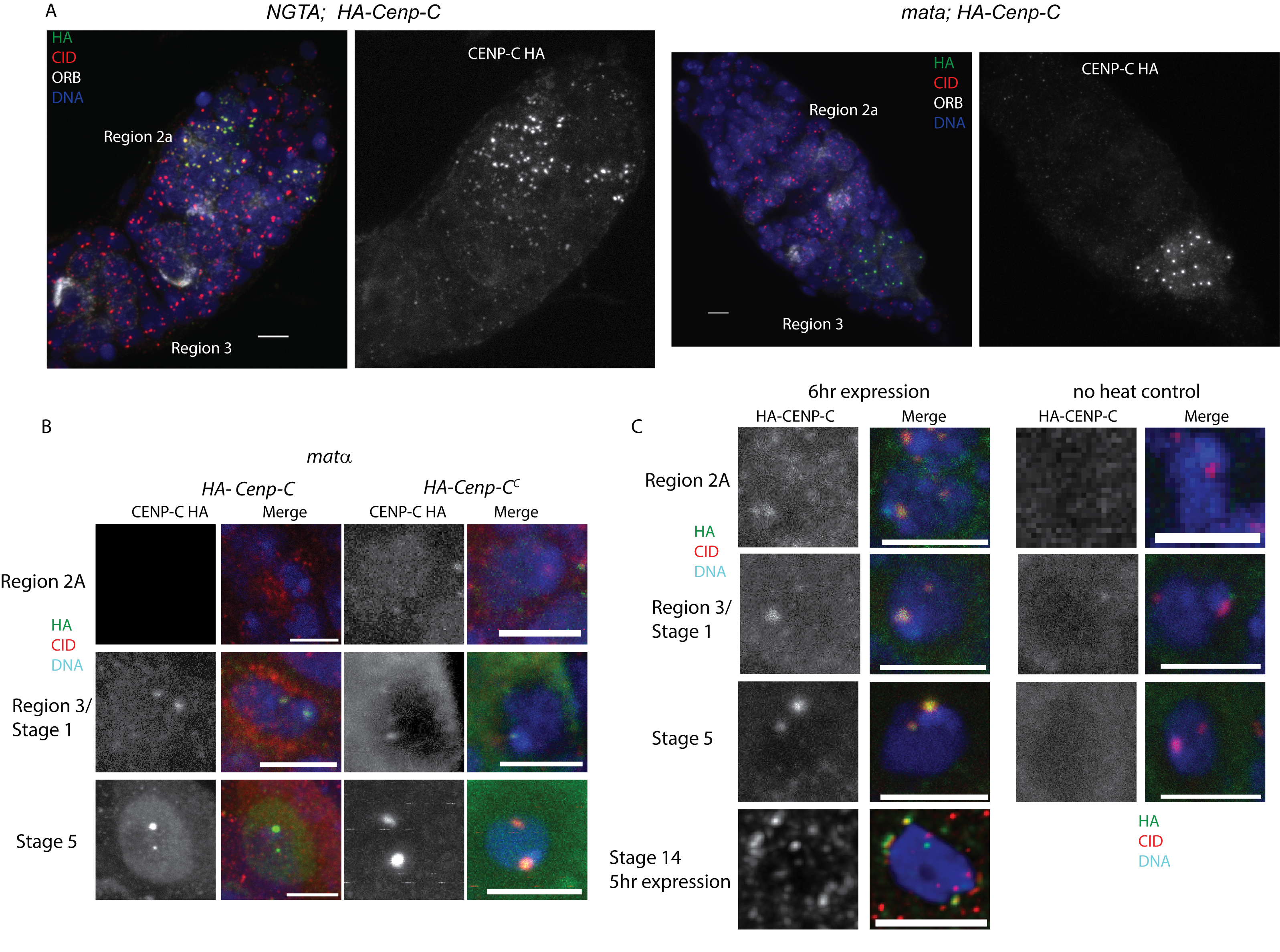

Supplement: S4 Fig — In all images, HA-CENP-C is green, the centromeres are marked with CID (red), and DNA is in blue. The scale bars represent 5 μm. A) Whole germarium with HA-tagged CENP-C expressed using NGTA or matα. ORB (white) is enriched in the oocyte. B) HA-tagged CENP-C or CENP-CC was expressed using matα. C) HA-CENP-C was expressed using hsp70-Gal4. Oocytes were collected and fixed after 6 hours (early prophase) or 5 hours (stage 14) after a 1-hour incubation at 37°C. (TIF) [file pgen.1011066.s004.tif]

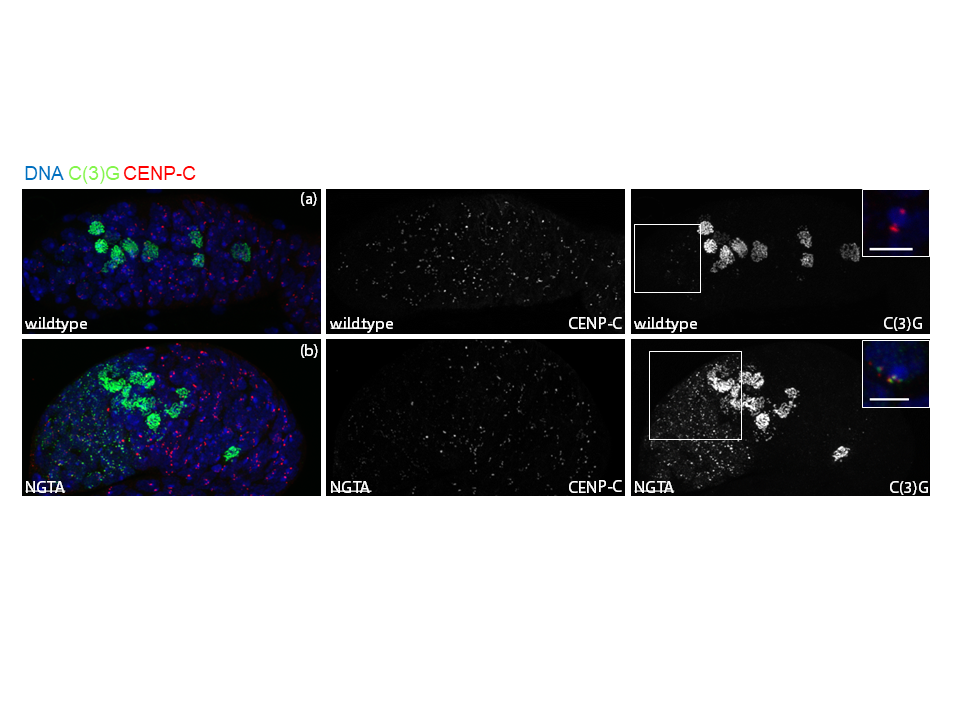

Supplement: S5 Fig — Confocal images of the germarium with Cenp-C RNAi (HMS01171) with (A) no GAL4 and (B) NGTA. DNA is shown in blue, CENP-C is in red, and C(3)G is in green. The scale bar is 10 μm. CENP-C and C(3)G are shown in white in the single channel images. Region 1 of the germarium has been boxed to show increased centromeric C(3)G. The insets show single nuclei from region 1 in the germarium to show co-localization of CENP-C and C(3)G (Scale bar = 3 μm). (TIF) [file pgen.1011066.s005.tif]

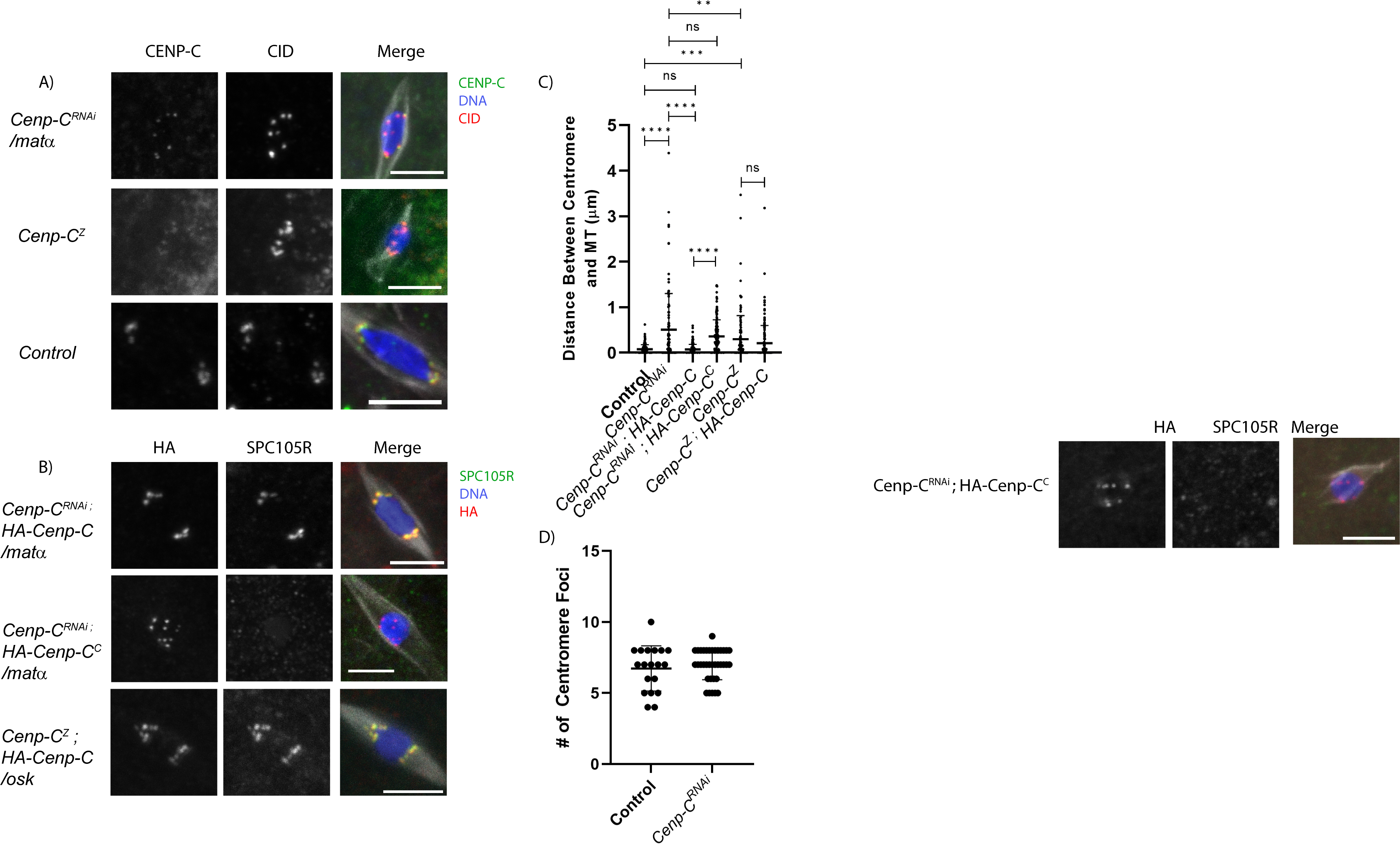

Supplement: S6 Fig — A) Cenp-C RNAi or Cenp-CZ oocytes with CENP-C (green) and CID (red). B) Cenp-C RNAi or Cenp-CZ oocytes expressing a Cenp-C transgene, with HA in red and Spc105R in green, DNA in blue, microtubules in white, and the scale bars represent 5 μm. C) Oocytes shown in panels A and B were assessed for KT-MT attachments. This was done by measuring the distance between each centromere and the nearest microtubule. D) Number of centromere foci was measured based on CID foci in Cenp-C RNAi metaphase I oocytes (n = 19 and 36). Error bars represent standard deviation. (TIF) [file pgen.1011066.s006.tif]

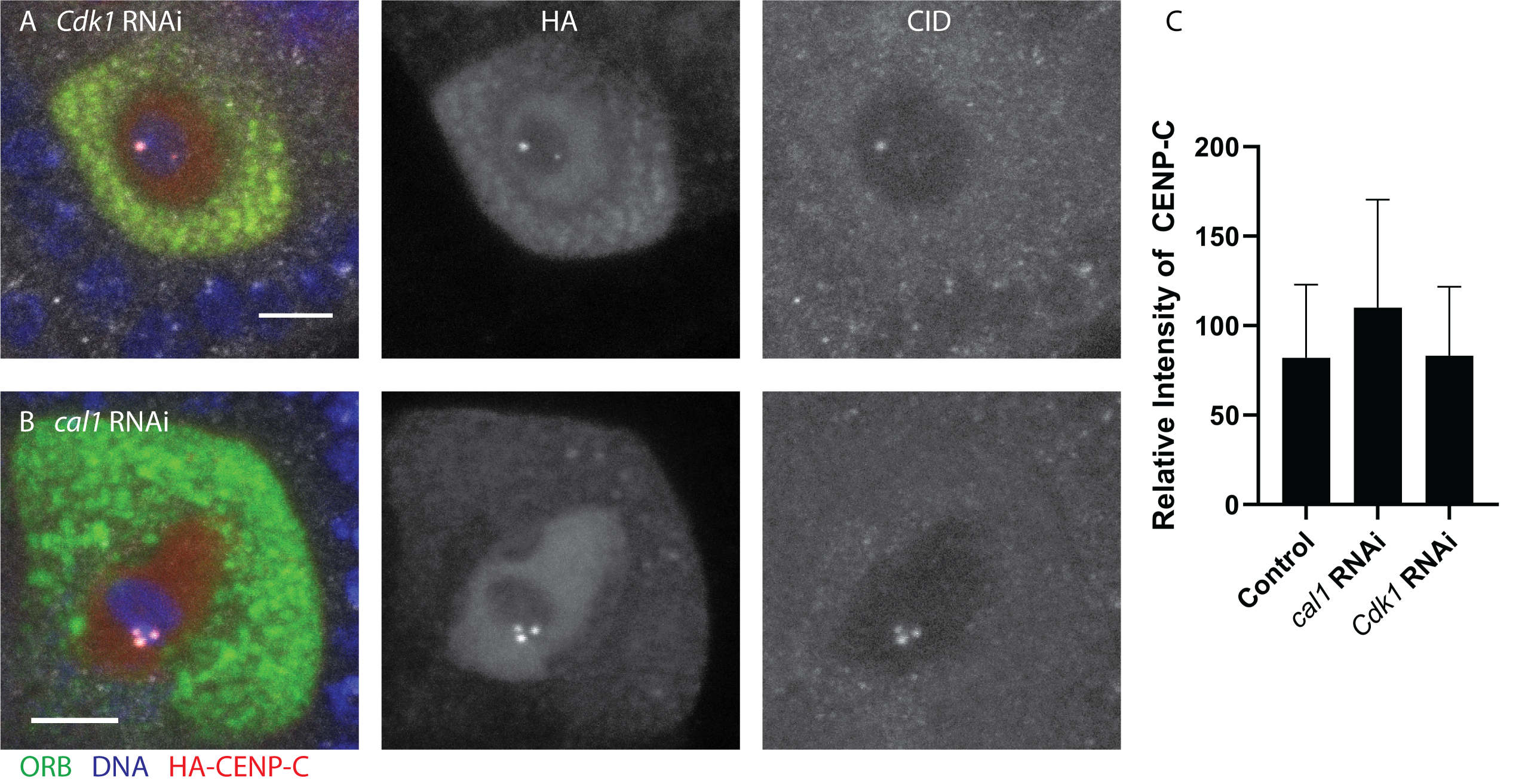

Supplement: S7 Fig — Localization of HA-CENP-C in stage 4–5 oocytes of (A) cdk1 and (B) cal1 RNAi oocytes. HA is in red, CID is in white, cytoplasmic ORB protein is in green, and DNA is in blue. The scale bar represents 5 μm. C) Relative intensity of CENP-C in control and RNAi oocytes (n = 18, 21, 18). (TIF) [file pgen.1011066.s007.tif]
