# Supplementary material for: A dynamic population of prophase CENP-C is required for meiotic chromosome segregation
Source: PLoS Genet. 2023 Nov 29;19(11):e1011066. doi: 10.1371/journal.pgen.1011066 (PMC10721191; doi:10.1371/journal.pgen.1011066)
Supplement: Attachment — Submitted filename: PLOSgenetics_Fellmeth_final.pdf [file pgen.1011066.s008.pdf]

This paper investigates the centromere recruitment dynamics of three key Drosophila proteins CID/CENP-A, CENP-C, and CAL1 in the female germ line. Using GFP-fusions exogenously expressed under three different germline drivers, the authors identify two distinct pools of GFP-CENP-C, one that is recruited to centromeres alongside CID and CAL1 in premeiotic and early prophase I phases, and another that is recruited during prophase I (and possibly later, see below). Using RNAi-mediated protein knock-downs, the authors show that, while the early wave of CENP-C is required for centromere clustering and loading of Mis12, the prophase wave is required for kinetochore assembly.

My major comments revolve around the interpretation of the data and their discussion in the context of previous work in the field, both of which fall short and need to be fully addressed before acceptance in PLoS Genetics.

1. Line 30-33: In the abstract the authors need to clearly demarcate where they switch from background information to their original findings. In addition, what's presented in this paper in terms of new findings needs to be framed in the context of previous work, specifically the finding that shows gradual CID loading during the meiotic prophase (Ref#48).
2. In the introduction, CID should be introduced alongside CENP-A since the term CID is used throughout the manuscript. Currently, the CID/CENP-A nomenclature is only mentioned in the abstract.
3. The introduction is not adequately detailed and lacks key information. Alongside mentioning work in other species the authors should pay special attention to work done in *Drosophila*. Even though many of the papers are listed in the references, the key findings from these papers are not appropriately acknowledged and mentioned. For example:
    A. **Lines 73 - 75** do not provide sufficient details to the reader about what is known about CID loading. CID loading timing in meiosis differs from that in mitosis and only mitosis is mentioned. Ref #45 shows that CID is gradually loaded at the centromeres throughout prophase I during oogenesis in *Drosophila*. Furthermore, Dattoli et al., 2020 investigated CENP-A dynamics in *Drosophila* GSCs, which is worth mentioning in the text.
    B. **Lines 78 - 79** should elaborate on how CID, CENP-C, and CAL1 maintain centromere identity. CAL1 is the functional homolog of HJURP in *Drosophila* and CAL1, CID and CENP-C are reciprocally interdependent for their localization and recruitment. The function of CAL1 as the assembly factor for CID is especially relevant to interpret some of the data in this paper (see below).
    C. It is worth mentioning work investigating the role of CENP-C deposition in centromere assembly in male meiosis spermatogenesis (Kwenda et al 2016). A brief examination of how this paper relates to the present work is also warranted in the discussion.

4. **Lines 204 - 206**: The authors should be explicit about the fact that CENP-C is required in the early pre-meiotic mitotic stages in the germ line because it is necessary for CID loading. This was shown by by Carty et al., 2021 and should be mentioned.
5. Throughout the paper, the authors need to make clear that they are expressing (and following) exogenously expressed GFP-CENP-C using different Gal4 drivers, while endogenous CENP-C (and CID and CAL1) are still present and expressed normally. They also should be explicit in which experiments exogenous GFP-CENP-C is present.
6. In lines **216 - 225 and Figure 3B,** the authors show only a mild decrease in centromeric CENP-C signal intensity, but a significant decrease in Mis12 localization (Figure 3D-E) in CENP-C knock-downs. Since CENP-C recruits Mis12, and it is still present at the centromeres in this experiment, then Mis12 levels

should not be affected. How do the authors explain this discrepancy? Furthermore, the authors should make clear that here they are knocking down and measuring endogenously expressed CENP-C (or are they? this point is not clear), since for other experiments they primarily follow exogenously expressed GFP-CENP-C.

7. **Lines 220 - 221**: Ref #20 and Hornung et al., 2014 show that Mis12 directly binds to CENP-C and should be cited for this statement.
8. Line 378 specify "when CENP-C was depleted in meiotic prophase".
9. When discussing the results of Figure 1 and in the abstract, the authors state that there is no CID loading in meiotic prophase I. However, their results could interpreted in a different way. The authors looked at EGFP-CID expression driven by the UAS-GAL4 system, which is distinct from endogenous CID levels. Expression of EGFP-CID and EGFP-CAL1 using NGTA and MVD1 GAL4 drivers shows that these GFP-tagged proteins localize at centromeres throughout oogenesis while, when expressed using mata GAL4, they are not present at the centromere at any stage in oogenesis. But we do not know **why** no GFP signal is detected in the mata experiments. Although these experiments show that CAL1 and CID expressed early in oogenesis are deposited at the centromere, they do not show that the loading of this early-expressed pool does not continue into prophase. In fact, the result that CENP-C RNAi in prophase (with mata) causes a small reduction of CID centromeric levels (Figure 3) would be consistent with defective CID loading in prophase. The authors mention their findings leave open the possibility of prophase loading (consistent with the data in Ref#48) in the discussion, but they do not sufficiently explain this alternative and reasonable explanation of their observation of the lack of GFP-CID loading in the discussion or the results. NGTA>EGFP-CID could be gradually deposited throughout prophase I, or perhaps it cannot be recruited at centromeres because there's no free CAL1 available (see next point for the lack of discussion and data on this critical point). Regardless, there could be endogenous CID from the early wave of expression still being deposited at the centromere (which would be consistent with the findings in Ref #48). To test this, GFP-CID or CID fluorescence intensity would need to be accurately measured at each stage because the difference is expected to be gradual. The authors need to at least explain their findings more critically and with more context in the results and discussion.
10. Along the lines of distinguishing endogenous versus exogenous CID expression timing and loading, the authors need to discuss their findings more in-depth throughout the results and in the discussion. How do they interpret the lack of GFP-CID and GFP-CAL1 signal at centromeres when these proteins are expressed starting in prophase I using the mata driver? Do they detect GFP expression by IF (even if not localizing to the centromere- is GFP fluorescence visible?) and Western blot, which would suggest the proteins are produced but the loading machinery is either missing or inhibited? Or are these proteins not even expressed, which would suggest they are degraded? CAL1 is the CID assembly factor in *Drosophila* (Ref#10). An explanation for the failure of GFP-CID to load in prophase I could be that endogenous CAL1 is not expressed at this time and thus there is no assembly factor available for loading. Soluble CAL1 expressed from the earlier stages could already be associated with endogenous CID (also expressed early) and if no new CAL1 is produced to couple with newly expressed GFP-CID, no loading with GFP-CID is possible. Similarly, since CID and CAL1 are reciprocally required for centromere loading, no new GFP-CAL1 can be loaded if endogenous CID from the earlier stages is all coupled up with endogenous CAL1. One way to partially address these different possibilities is to co-express GFP-CID and GFP-CAL1 (even better would be to use different tags) with mata and see if the GFP-CID and GFP-CAL1 centromeric recruitments in prophase I are rescued. Even without this experiment, the authors must more critically explain and discuss their data and these plausible interpretations.

11. The novel finding in this paper is that there are two CENP-C pools with different functions in *Drosophila* oogenesis. Right from the beginning of the paper, the authors need to make a clear distinction by using precise terminology for these two pools of CENP-Cs (e.g. premeiotic pool and prophase I pool) as well as explicitly distinguishing these exogenously expressed GFP-CENP-C pools from the already centromeric endogenous CENP-C. This clarification will help readers understand what is known from previous studies and what are the novel findings in this paper. For example, in lines 198 - 199, the header states that CENP-C regulates centromere clustering and recombination, but this is not a new result, it was previously shown in Ref #47 and the authors do not mention this study in this context. The authors add to this work by showing that **only one** of the two CENP-C pools is involved in centromere clustering and recombination. They should also explicitly acknowledge this previous study and findings in the results lines 252 - 254.

Minor Comments:

1. Mis12 is incorrectly written as MIS12 and sometimes MIS-12.
2. In all the figure legends, the scale bar is written as ‘u’m instead of ‘µ’m.
3. Figure 1E is pixelated, and thus hard to read. Similarly, graphs in most of the figures need to be correctly re-inserted in the figure such that they have better resolution, the labels are barely readable in some cases.
4. In Figure 2E, the X-axis across all three graphs should be kept consistent.
5. SPC105R needs to be introduced and the proper rationale is needed for looking at this protein.
6. Line 280-281 "CENP-C plays a direct role in error correction, but more likely has an indirect effect due to the function of CENP-C in kinetochore assembly." There are multiple published studies that support the second possibility, the authors should include at least some key papers that are consistent with this conclusion.
7. Line 326-328 "These results support the conclusion that the C-terminal domain of CENP-C is required for centromere localization, while the N-terminal domain promotes recruitment of MIS12 and the rest of the kinetochore" I think it should be made clear that the latter is a logical inference as no data directly show this.
8. For lines 365-366, please provide citations instead of referring back to the introduction, where the reader can’t find more in-depth information than is in this sentence.
