# Supplementary material for: A dynamic population of prophase CENP-C is required for meiotic chromosome segregation
Source: PLoS Genet. 2023 Nov 29;19(11):e1011066. doi: 10.1371/journal.pgen.1011066 (PMC10721191; doi:10.1371/journal.pgen.1011066)
Supplement: Attachment — Submitted filename: Responses.pdf [file pgen.1011066.s009.pdf]

Comments to the Authors:

Reviewer #1: The paper by Fellmeth et. al. addresses the roles of centromeric proteins CENP-C, CENP-A/CID and the loader CAL1 in the Drosophila oogenic gremline. The paper utilized powerful genetic tools to dissect early and late meiotic functions. They discover that early expression of CID is sufficient for its localization to centromeres in oocytes and restricting its meiotic accumulation may be important for suppressing ectopic centromeres. Their studies propose that CENP-C is constantly loaded throughout prophase, with early prophase loading important for centromeric cohesion. Late loading of CENP-C is required for kinetochore protein recruitment. The studies are well done and, generally speaking, clearly presented. I have some suggestions for improving the text and figures in a way that will be accessible to readers outside the first field of study.

• Introduction: key information that is missing her is the localization pattern of CID and CENP-C in Drosophila oogenesis. There is a discussion of their mitotic localization, but there is no clear statement regarding Drosophila oogenesis. This information is already published (e.g., Fellmeth and Kim 2020 Fig 1) and is important for understanding the experiments presented. In addition, it is important to indicate it in the introduction (and not wait for the discussion) that CID progressively assembles during meiotic prophase (Dunleavy 2012).

References to published descriptions of the localization patterns for CID and CENP-C in Drosophila oogenesis have been added to the Introduction (lines 90-96).

• The description of experiments could be improved to facilitate understanding of the experimental design by the general (scientific) public. I suggest the following points explained in more details:
o Line 117-125: I assume that all the experimental conditions in Figure 1 are UASP-driven expression in the background that contains the endogenous genes. It should be stated clearly. The reader should be informed early on that this can lead to overexpression (for all conditions and not just CID).

We have added a statement in lines 137-138 that UASP -driven expression is in addition to endogenous and therefore, possibly overexpression. A similar statement was added to the Figure 1 legend.

o Line 176-182: clarify that all endogenous CENP-C is GFP tagged

None of the experimental conditions have the endogenous Cenp-C gene tagged with GFP. In some experiments, there is an extra copy of a Cenp-C-GFP transgene inserted into the genome with its own promoter. This has been described in the text on lines 201-203.

o Line 201-202: The authors move from describing driving protein to driving shRNA expression and if the reader misses that, then the whole next section is very confusing. "To investigate Cenp-C function in early prophase, we knocked down its expression by RNAi using an shRNA (GL00409) with either the NGTA or MVD1 promoters." I would expand the description here (and break to 2-3 sentences) to specifically state that Cenp-c is the endogenous copy and that the shRNA driven from MVD1 will result in knockdown of cenp-c throughout the germline and shRNA driven from NGTA will result in knockdown of cenp-c early in meiosis.

RUTGERS
NEW BRUNSWICK

Expanded description of the experiment with multiple sentences to explain that endogenous Cenp-C is knockdown with MVD1 in all germline stages (lines 232-234) or NGTA in meiosis (lines 238-239).

• The quality of the graphs is very low on this version. It was very hard to understand them as the axis labeling was frequently pixelated. This must be fixed for publication. Figure 1E should be enlarged. I assume that in publication it won't take the whole page and therefore will be smaller (and unreadable the way it is now).

All graphs were replaced with higher resolution versions. They tend to look better in tiff files than pasted into word docs.

• If indeed all experiments in Figure 1 were done in the background of wild type copy of the protein, then these are also over expression conditions. It is not clear why overexpression effect on localization is only considered for CAL1 and therefore only for it a control experiment is performed. Including the localization patten of CID and CENP-C driven by endogenous promoter in Fig 1 will help. The endogenous protein localization pattern should be shown in Figure 1 (for stage 1 and 14), so it can be compared to the stage-specific expression. There is some data about that for CAL1 in S2 but not for earlier stages.

We discuss overexpression generally (lines 137-138) and specifically for CAL1 (lines 153-155) and CID (lines 149-150, 169-171). The latter leads to incorporation throughout the chromatin. The control (gCal1) was performed because of the absence of CAL1 in metaphase I oocytes and we do not have a CAL1 antibody. We have added a sentence to make this point (lines 152-153). We used antibodies for CID (Figure 1) and CENP-C (Figure 1) to detect endogenous protein and be centromere markers in several experiments. The intensity of each antibody is noted in Figure 3 B&C and Figure 5 B&C. We have kept localization of gCAL1 in Supp Fig 1 because Fig 1 is already too crowded.

• The organization of figure 1 is confusing and is not in line with the text. The text discuses MVD1 before NGTA but the figure order is reversed. Same is with graph in the figure

We have changed the organization of Figure 1 and text in Results so that they match. The order is MVD1 first, then mata and NGTA, and within each the order is CENP-C, CID and CAL1.

• The description of the CID-MVD1 (line 129-131) needs to be rewritten in a way the reflects figure 1E. The way it reads now is as if there are only 2 categories

We have made the categories in the graph match the text. In the graph we merged some categories. Notably, "GFP on chromosomes" was merged with "GFP on chromosomes and centromeres" because it was difficult to distinguish these two and the important thing is the abnormal chromatin CID localization.

• Figure 2E- the order on the x axis should be the same between the graphs. The labeling of the X axis should be explained in the figure legend. I understand it's a shorter version of B-D but it is confusing since its different. the Y axis should be the same in all graphs in E.

The order of the X-axis is now the same between graphs. In addition, the abbreviations are now defined in the Figure)

• The authors need to explain why loss of cohesion leads to increase in crossover. Do they see more centromeric DSBs or is this a result of a change in CO/NCO decision post-DSB formation?

Our writing was confusing. The assay we performed to measure CO's is based on the phenotypes of offspring. We did not measure DSB's or NCO's. This was rewritten and we have added references that suggest loss of pericentric cohesion affects crossover distribution (lines 274-275).

• Figure 3-5 the RNAi is stage-specific and must be differentiated by introducing different naming for each RNAi condition in the figure panels. Just saying RNAi implies it's all over the organism.

The panels if Figures 3-5 now have labels for the GAL4 being used.

• Discussion: CID localization is only partially dependent on CENP-C and its overexpression is sufficient for formation of ectopic chromatin-associated foci. Is it possible there is a CENP-C independent mechanism for CID recruitment? This option should be discussed.

We agree this is an important point. We added a sentence on pg 436-438 that there might be a CENP-C independent mechanism.

The authors show here that CAL1 is unloaded in prophase. Dunleavy 2012 showed that CID levels are increased during female prophase. CAL1 is also a chaperon loader of CID. It is therefore unclear what loads CID in prophase. Can this be discussed?

We have added Discussion of the Dunleavy 2012 results, although our data regarding CID loading mechanisms is limited. There is an important difference in staging between these two experiments. We observe loss of CAL1 between stage 5 and 14. Stage 8/9 was the last measured in Dunleavy 2012. Thus, CAL1 is present in our experiments during the time Dunleavy 2012 observed loading of CID. That is, some or most of our observed CAL1 loss could be after the measurements of Dunleavy 2012 (lines 428-429).

• Discussion: Page 23, Line 434 the authors cite mammalian papers to say that CENP-A/CID is not loaded in prophase. However, in Drosophila oocytes (Dunleavy 2012, Figure 3B) CID continuously loads. Related- Page 20, line 379: Why do the others state that the loading of CID in prophase is small? I understand the argument that restricted loading is fine but high levels of lodging are detrimental to oogenesis, but unless the data in Dunleavy 2012 is incorrect I don't think the authors should downplay prophase loading of CID.

Our results are consistent with mammalian studies in that we show that CID loaded early is stable (as opposed to Cenp-C and CAL1). We have discussed the differences between our results and Dunleavy on pages 21-22). However, the two experiments are different. We tested if early prophase loading (based on NGTA) was sufficient in the absence of prophase loading. The results for CID and CENP-C are dramatically different. Dunleavy 2012 results indicate CID loads in prophase, although it is not known if this is required for meiosis. Both could be true. Our experiment with CID mata did not address this question because of overexpression and resulting ectopic CID localization (Figure

1, Figure S1). We have made edits to try and eliminate any downplaying of these previous results. In addition, we deleted the comment about "small amount of CID prophase loading".

• A model describing the findings of this paper and comparing them to mammalian systems should be added.

We added a model as Figure 8.

Reviewer #2:

My major comments revolve around the interpretation of the data and their discussion in the context of previous work in the field, both of which fall short and need to be fully addressed before acceptance in PLoS Genetics.

1. Line 30-33: In the abstract the authors need to clearly demarcate where they switch from background information to their original findings. In addition, what's presented in this paper in terms of new findings needs to be framed in the context of previous work, specifically the finding that shows gradual CID loading during the meiotic prophase (Ref#48).

   Added a sentence (lines 29-30) to indicate where the description of our findings begins. Regarding our findings in the context of previous work, that is addressed below.

2. In the introduction, CID should be introduced alongside CENP-A since the term CID is used throughout the manuscript. Currently, the CID/CENP-A nomenclature is only mentioned in the abstract.

   In line 68 indicated that CENP-A is known as CID in Drosophila.

3. The introduction is not adequately detailed and lacks key information. Alongside mentioning work in other species the authors should pay special attention to work done in *Drosophila*. Even though many of the papers are listed in the references, the key findings from these papers are not appropriately acknowledged and mentioned. For example:
   1. **Lines 73 - 75** do not provide sufficient details to the reader about what is known about CID loading. CID loading timing in meiosis differs from that in mitosis and only mitosis is mentioned. Ref #45 shows that CID is gradually loaded at the centromeres throughout prophase I during oogenesis in *Drosophila*. Furthermore, Dattoli et al., 2020 investigated CENP-A dynamics in *Drosophila* GSCs, which is worth mentioning in the text.

      We have added more background information and references for the describing the mechanism of CID loading (lines 90-96), including the differences observed in the Drosophila germlines.

   2. **Lines 78 - 79** should elaborate on how CID, CENP-C, and CAL1 maintain centromere identity. CAL1 is the functional homolog of HJURP in *Drosophila* and CAL1, CID and CENP-C are reciprocally interdependent for their localization and recruitment. The

function of CAL1 as the assembly factor for CID is especially relevant to interpret some of the data in this paper (see below).

We have added a description for how these three proteins maintain centromere identify in lines 83-89.

3. It is worth mentioning work investigating the role of CENP-C deposition in centromere assembly in male meiosis spermatogenesis (Kwenda et al 2016). A brief examination of how this paper relates to the present work is also warranted in the discussion.

   Reference to Kwenda was added in the Introduction (lines 93, 96) and Discussion (lines 419, 442 and 457).

4. **Lines 204 - 206**: The authors should be explicit about the fact that CENP-C is required in the early pre-meiotic mitotic stages in the germ line because it is necessary for CID loading. This was shown by by Carty et al., 2021 and should be mentioned.

   Added comment about pre-meiotic mitotic stages and referenced Carty 2021 in line 235.

5. Throughout the paper, the authors need to make clear that they are expressing (and following) exogenously expressed GFP-CENP-C using different Gal4 drivers, while endogenous CENP-C (and CID and CAL1) are still present and expressed normally. They also should be explicit in which experiments exogenous GFP-CENP-C is present.

   See also Reviewer 1. We have made changes in several places (lines 137-138, 149-150, 153-155, 169-171, 201-202) to make it clear when the expression of tagged cen proteins is being monitored while endogenous proteins are still present and possibly overexpressed. We also made changes to use the genotytpe "gGFP-Cenp-C" for the version of exogenous Cenp-C that is GFP tagged but regulated by its own promoter.

6. In lines **216 - 225 and Figure 3B,** the authors show only a mild decrease in centromeric CENP-C signal intensity, but a significant decrease in Mis12 localization (Figure 3D-E) in CENP-C knock-downs. Since CENP-C recruits Mis12, and it is still present at the centromeres in this experiment, then Mis12 levels should not be affected. How do the authors explain this discrepancy? Furthermore, the authors should make clear that here they are knocking down and measuring endogenously expressed CENP- C (or are they? this point is not clear), since for other experiments they primarily follow exogenously expressed GFP-CENP-C.

   We added an explanation in lines 262-264 that this result is consistent with two populations of CENP-C. We have also added text (lines 231-239) that endogenous CENP-C is being knocked down. We are measuring endogenous CENP-C and it is measured with CENP-C antibody.

7. **Lines 220 - 221**: Ref #20 and Hornung et al., 2014 show that Mis12 directly binds to CENP-C and should be cited for this statement.

   These citations were added in lines 258.

8. Line 378 specify "when CENP-C was depleted in meiotic prophase".

This sentence was corrected (line 423).

9. When discussing the results of Figure 1 and in the abstract, the authors state that there is no CID loading in meiotic prophase I. However, their results could interpreted in a different way. The authors looked at EGFP-CID expression driven by the UAS-GAL4 system, which is distinct from endogenous CID levels. Expression of EGFP-CID and EGFP-CAL1 using NGTA and MVD1 GAL4 drivers shows that these GFP-tagged proteins localize at centromeres throughout oogenesis while, when expressed using mata GAL4, they are not present at the centromere at any stage in oogenesis. But we do not know **why** no GFP signal is detected in the mata experiments. Although these experiments show that CAL1 and CID expressed early in oogenesis are deposited at the centromere, they do not show that the loading of this early-expressed pool does not continue into prophase. In fact, the result that CENP-C RNAi in prophase (with mata) causes a small reduction of CID centromeric levels (Figure 3) would be consistent with defective CID loading in prophase. The authors mention their findings leave open the possibility of prophase loading (consistent with the data in Ref#48) in the discussion, but they do not sufficiently explain this alternative and reasonable explanation of their observation of the lack of GFP-CID loading in the discussion or the results. NGTA>EGFP-CID could be gradually deposited throughout prophase I, or perhaps it cannot be recruited at centromeres because there's no free CAL1 available (see next point for the lack of discussion and data on this critical point). Regardless, there could be endogenous CID from the early wave of expression still being deposited at the centromere (which would be consistent with the findings in Ref #48). To test this, GFP-CID or CID fluorescence intensity would need to be accurately measured at each stage because the difference is expected to be gradual. The authors need to at least explain their findings more critically and with more context in the results and discussion.

   We have tried to remove any insinuation that there is no CID prophase loading or localization using mata. Our conclusion is that high levels of expression are deleterious (line 432-438), which could be a reason to limit prophase expression of CID. The next conclusion is that NGTA (regions 1-3) expression is sufficient for stage 14 localization, while the same is not true for CENP-C. We have added (lines 180-181) the caveat that mRNA persists from the early stages to allow gradual CID expression and deposition in the later stages of oogenesis. It seems more likely that CID is like cohesins, where NGTA expression is sufficient for stage 14 function because the protein localizes to the chromosomes early in oogenesis.

   We discuss similarities and differences between among our findings and Dunleavy 2012 (lines 418-431). Primarily, Dunleavy 2012 showed evidence of prophase loading based on changes in immunofluorescence intensity. We have also shown some changes by a similar assay (line 422-423), although this was in Cenp-C RNAi and there are other interpretations, such as loss of loaded CID because CENP-C is lost. As noted above, we have shown that early expression is sufficient for CID localization in late oocyte stages. This leaves open the possibility that significant amounts of CID are retained while some amounts are also loaded. The function of meiotic prophase loading of CID is, to our knowledge, not known. Another source of difference could be the stages analyzed. The endpoint for Dunleavy 2012 (Figure 3) is stage 8/9, which we did not examine. Our datapoint where Cal1 is absent is stage 14, which Dunleavy did not measure. Thus, the loading that Dunleavy observed could be occurring during the same stages where we still see Cal1 localized to the centromeres (lines 428-430).

10. Along the lines of distinguishing endogenous versus exogenous CID expression ,timing and loading, the authors need to discuss their findings more in-depth throughout the results and in the

discussion. How do they interpret the lack of GFP-CID and GFP-CAL1 signal at centromeres when these proteins are expressed starting in prophase I using the mata driver? Do they detect GFP expression by IF (even if not localizing to the centromere- is GFP fluorescence visible?) and Western blot, which would suggest the proteins are produced but the loading machinery is either missing or inhibited? Or are these proteins not even expressed, which would suggest they are degraded? CAL1 is the CID assembly factor in *Drosophila* (Ref#10). An explanation for the failure of GFP-CID to load in prophase I could be that endogenous CAL1 is not expressed at this time and thus there is no assembly factor available for loading. Soluble CAL1 expressed from the earlier stages could already be associated with endogenous CID (also expressed early) and if no new CAL1 is produced to couple with newly expressed GFP-CID, no loading with GFP-CID is possible. Similarly, since CID and CAL1 are reciprocally required for centromere loading, no new GFP-CAL1 can be loaded if endogenous CID from the earlier stages is all coupled up with endogenous CAL1. One way to partially address these different possibilities is to co-express GFP-CID and GFP-CAL1 (even be\er would be to use different tags) with mata and see if the GFP-CID and GFP-CAL1 centromeric recruitments in prophase I are rescued. Even without this experiment, the authors must more critically explain and discuss their data and these plausible interpretations.

As noted above, CID is observed with mata, but it is abnormal. For CAL1, which is absent from centromeres in late stage oocytes, we have added evidence of mata expression based on staining in the cytoplasm (Supp Fig 1D), and localization to the spindle in mature oocytes (Figure 1C). In general, we are confident that the CID-GFP and CAL1-GFP constructs are being expressed because we observe the localization of these constructs with other GAL4 drivers. We agree that a CAL1-CID co-expression experiment would be interesting, but is beyond the scope of this paper which is more about CENP-C. We have made changes (noted above) to more critically explain our data and interpretations.

11. The novel finding in this paper is that there are two CENP-C pools with different functions in *Drosophila* oogenesis. Right from the beginning of the paper, the authors need to make a clear distinction by using precise terminology for these two pools of CENP-Cs (e.g. premeiotic pool and prophase I pool) as well as explicitly distinguishing these exogenously expressed GFP-CENP-C pools from the already centromeric endogenous CENP-C. This clarification will help readers understand what is known from previous studies and what are the novel findings in this paper. For example, in lines 198 - 199, the header states that CENP-C regulates centromere clustering and recombination, but this is not a new result, it was previously shown in Ref #47 and the authors do not mention this study in this context. The authors add to this work by showing that **only one** of the two CENP-C pools is involved in centromere clustering and recombination. They should also explicitly acknowledge this previous study and findings in the results lines 252 - 254.

This is an excellent suggestion, and we have used the terms "premeiotic" and "prophase I" pool (line 229-231) and added text to help the reader distinguish these pools.

The reference for CENP-C regulating centromere clustering is added in line 292-294 and cited in the Discussion (line 455).

Minor Comments:

1. Mis12 is incorrectly written as MIS12 and sometimes MIS-12.

Corrected throughout the manuscript.

2. In all the figure legends, the scale bar is written as ‘u’m instead of ‘μ’m.

Corrected in all Figure legends.

3. Figure 1E is pixelated, and thus hard to read. Similarly, graphs in most of the figures need to be correctly re-inserted in the figure such that they have better resolution, the labels are barely readable in some cases.

Higher resolution graphs were inserted into all Figures.

4. In Figure 2E, the X-axis across all three graphs should be kept consistent.

   Figure 2E X-axis labels are now consistent among the graphs.

5. SPC105R needs to be introduced and the proper rationale is needed for looking at this protein.

   Kinetochore protein introduced on pages 166 and 305.

6. Line 280-281 "CENP-C plays a direct role in error correction, but more likely has an indirect effect due to the function of CENP-C in kinetochore assembly." There are multiple published studies that support the second possibility, the authors should include at least some key papers that are consistent with this conclusion.

   Added citations (line 322-323) consistent with the second possibility.

7. Line 326-328 "These results support the conclusion that the C-terminal domain of CENP-C is required for centromere localization, while the N-terminal domain promotes recruitment of MIS12 and the rest of the kinetochore" I think it should be made clear that the latter is a logical inference as no data directly show this.

   The was edited. Because a version of CENP-C without its N-terminal domain lacks Spc105R and Mis12, the data suggests this part of CENP-C recruits outer KT proteins (lines 367-369).

8. For lines 365-366, please provide citations instead of referring back to the introduction, where the reader can’t find more in-depth information than is in this sentence.

   Citations added to line 407.
